# Orofacial Cat Bite: A Case Report

**DOI:** 10.5005/jp-journals-10005-1031

**Published:** 2009-08-26

**Authors:** Preetika Chandna, Vivek Kumar Adlakha, Manisha Prabhakar, Sanjeev Julka

**Affiliations:** 1 Lecturer, Department of Pedodontics and Preventive Dentistry, Subharti Dental College, Meerut, Uttar Pradesh, India; 2 Lecturer, Department of Pedodontics and Preventive Dentistry, Subharti Dental College, Meerut, Uttar Pradesh, India; 3 Professor and Head, Department of Pedodontics and Preventive Dentistry, IDST Dental College, Kadrabad, Modinagar Uttar Pradesh, India; 4 Department of Pedodontics and Preventive Dentistry, Jodhpur National University, Jodhpur, Rajasthan, India

**Keywords:** Cat bite, orofacial trauma.

## Abstract

The article describes an unusual case of a 7 years old male child presenting with orofacial cat bite injury that occurred in infancy. This resulted in loss of deciduous and permanent tooth buds and consequently hampered alveolar growth. The patient was given a removable partial denture to restore function and aesthetics. The article highlights the importance of complete history, diagnosis and management of such injuries in children.

## INTRODUCTION

The primary objective of diagnosis and treatment of traumatic injuries affecting children in the primary dentition is the prevention of damage to the developing permanent dentition. Traumatic injuries in the form of animal bites may occur in a variety of circumstances, ranging from unprovoked attacks in the wild by naturally aggressive animals to injuries inflicted by household pets who are disturbed for any reason. Children are most common victims, particularly of dog bites.^1^ From birth, a child is exposed to episodes of traumatic injuries to the orofacial region which may range in severity from minor to life-threatening. However, incidents such as animal bites can have a number of temporary and long-term sequelae such as psychologic effects to the developing psyche, physical injury or impairment of function.^2^

The purpose of this paper is to illustrate the clinical findings in a 7 years old male patient presenting with orofacial cat bite injury.

## CASE REPORT

A 7 years old healthy male child reported to the out patient Department of Pedodontics and Preventive Dentistry, Christian Dental College, Ludhiana, India with chief complaint of noneruption of teeth, since birth, in the lower anterior region. A detailed history elicited from the mother of the child revealed an episode of assault to the child, by a cat, at about 6-8 months of age. The child apparently lay asleep in the veranda when he was attacked by a cat. The child was rescued by his mother on being alerted by his cries. However, by this time he had been severely bitten on the face. The child sustained multiple lacerations on the face and a part of the alveolus was bitten off. He was rushed to a local hospital where the bleeding was controlled and wounds sutured. Following this, no further medical or dental treatment was undertaken. Further questioning to rule out a possible history of hypodontia in the patient’s family was noncontributory. The patient’s medical history was unremarkable.

**Fig. 1:**
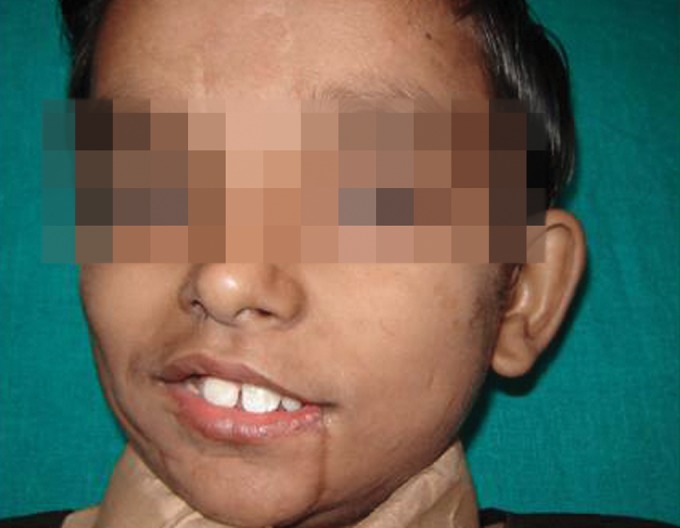
Extraoral photograph—Front view of patient’s face

**Fig. 2:**
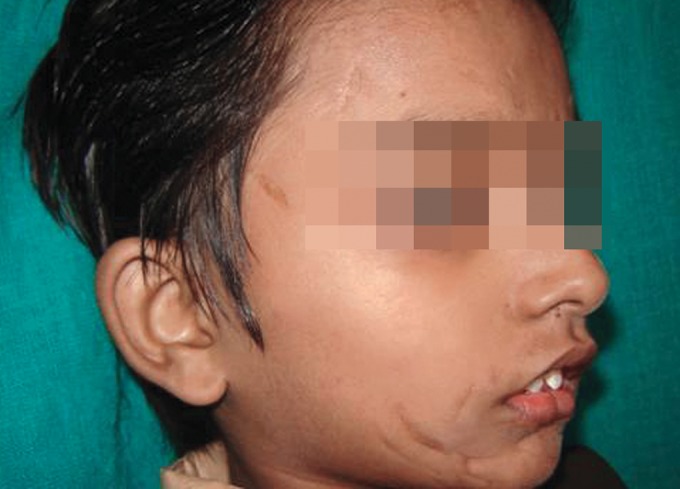
Extraoral photograph—Right view of patient’s face

**Fig. 3:**
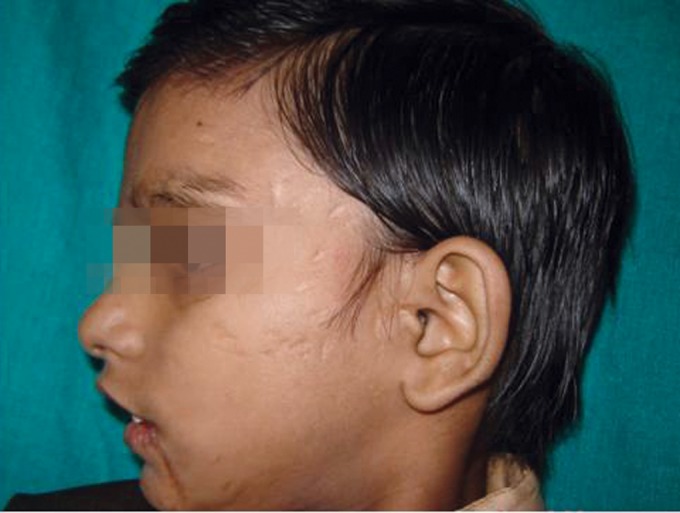
Extraoral photograph—Left view of patient’s face

**Fig. 4:**
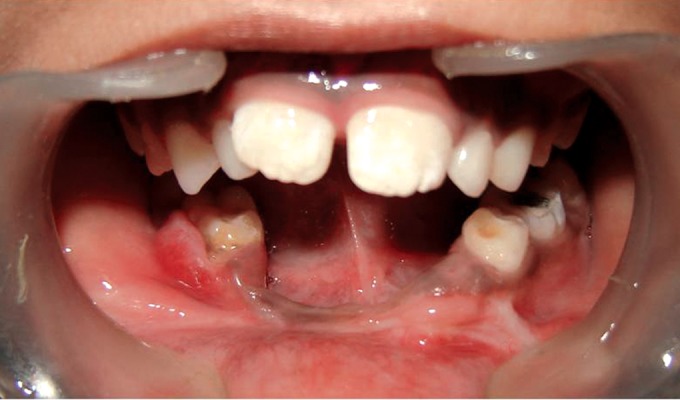
Intraoral photograph

**Fig. 5:**
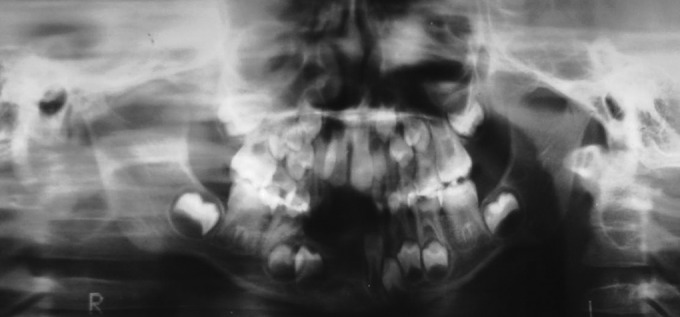
OPG X-ray

Extraoral examination revealed multiple scar marks on the supraorbital area, cheeks and perioral area (Figs 1 to 3). Intraoral examination revealed apparently missing teeth #73, 72, 71, 81, 82, 83 and 84 (Fig. 4). Another interesting finding was severe alveolar ridge resorption in the mandibular anterior ridge area. To confirm the clinical picture, an OPG was taken. The OPG revealed missing tooth buds of teeth #31, 41, 42 and 43 (Fig. 5).

**Fig. 6:**
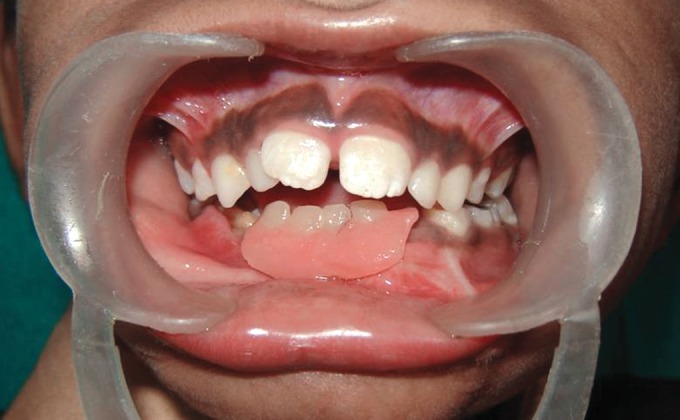
Removable partial denture in position

Despite the traumatic incident, the child remained cheerful and cooperative throughout examination and treatment, without any apparent emotional or psychological side effects. Treatment undertaken was directed towards maintaining arch length integrity, restoring aesthetics and avoidance of future speech defects. A polysiloxane (putty) impression was made of the upper and lower arches and models poured in dental stone. Following this, an acrylic removable partial denture was constructed and delivered (Fig. 6). The patient was recalled in a month’s time for review.

## DISCUSSION

Children of all ages are susceptible to traumatic injuries, ranging from minor to life-threatening ones. In case of animal attacks, this is more so^2^, because of their relatively decreased ability to protect themselves from injury due to their small size and strength as compared to adults. Another reason is their curiosity and playful nature that may provoke animals to attack them. Dog and cat bites are particularly more serious in children than in adults because children are more likely to be bitten on the face, neck and head in up to 70% of cases.^3^,^4^

Injuries due to animal attacks can result in short-term or long-term dental, medical and psychological effects. These may include mutilating injuries, disability or emotional side effects. There are scanty reports of animal bite injuries to the orofacial region. Williams BJ published a review and case report of orofacial dog bites detailing the role of the pediatric dentist.^2^ Dental follicle infection of primary maxillary canine following a dog bite has also been reported.^5^ There are no other reports of dog bite injuries to orofacial region. To the best of our knowledge, cat bite injuries to orofacial region has not been reported so far.

In the present case report, tooth eruption was absent in the anterior mandibular arch. It was suspected that the injury to the child due to the cat attack could have resulted in the loss of a chunk of alveolar ridge that contained deciduous and permanent tooth buds. Since the attack took place in the native village of the child, in the outskirts of the city, the bite was presumably due to a wild cat. Keeping in mind the child’s young age (6-8 months), the attack was probably unprovoked too.

Management of animal bite injuries involves securing a detailed history initially. A detailed history must be elicited and the animal and location of injury identified. Following this, the immunization status is assessed. Extent of injury is estimated and emergency treatment carried out such as wounds debridement and suturing if necessary.^2^ Pediatric dentists can further assess the psychological impact of the traumatic episode on the child through his or her reactions and general behavior. If needed, sedation may be given for emergency management.^2^

Animal bites represent a significant source of wound infection in humans.^6^ Saliva of many animals contains a wide variety of bacteria, predominantly *Bacteroides species, Pasteurella multocida and Porphyromonas* species which can result in fatal complications such as cellulitis, meningitis, lung abscess, cat scratch disease, tetanus or rabies.^7^-^9^ Crushing and puncture injuries and hand injuries have the highest potential for infection. Cat bites tend to be penetrating wounds and scratch injuries, with a higher risk for certain infections.^4^

Controversies remain about the use of antibiotics and the best way of avoiding infections after an animal bite. Management of infection can be divided into cleansing of the wound, antibiotic prophylaxis, and antibiotic treatment. The following recommendations have been suggested for the management of these injuries:^1^

1. Immediate wound cleansing with peroxide and saline is essential. Deep injuries should be rinsed by a syringe with a needle.
2. Primary wound closure with minimal debridement is advised. Infected wounds should be closed primarily after insertion of a drain.
3. Antibiotic prophylaxis after the bite is obligatory for all wounds of Lackmann class II and more. Children should generally be given antibiotics. Antibiotic prophylaxis should be continued for at least 5 days.
4. Antibiotic prophylaxis is necessary in all patients with wounds older than 6 hours and also in the presence of comorbidities such as immunosuppression, prosthetic heart valves, or diabetes.
5. After gram stains of dog bite wounds amoxycillinâ clavulanic acid by mouth (875 mg + 125 mg twice a day in adults) is the recommended antimicrobial agent.
6. Tetanus and rabies immunization history must be verified and vaccination and immune globulin must be given when indicated.
7. In all cases of cat bites, prophylaxis with amoxycillin clavulanate is necessary.
